# Childcare Food Provision Recommendations Vary across Australia: Jurisdictional Comparison and Nutrition Expert Perspectives

**DOI:** 10.3390/ijerph17186793

**Published:** 2020-09-17

**Authors:** Alison Spence, Penelope Love, Rebecca Byrne, Amy Wakem, Louisa Matwiejczyk, Amanda Devine, Rebecca Golley, Ros Sambell

**Affiliations:** 1 Institute for Physical Activity and Nutrition, Deakin University, Geelong, VIC 3216, Australia; penny.love@deakin.edu.au; 2 Institute of Health and Biomedical Innovation, Centre for Children’s Health Research, Queensland University of Technology, Brisbane, QLD 4000, Australia; ra.byrne@qut.edu.au; 3 Nutrition Australia Vic, Carlton, VIC 3053, Australia; AWakem@nutritionaustralia.org; 4 College of Nursing and Health Sciences, Flinders University, Adelaide, SA 5001, Australia; louisa.matwiejczyk@flinders.edu.au; 5 School of Medical and Health Sciences, Edith Cowan University, Joondalup, WA 6027, Australia; a.devine@ecu.edu.au (A.D.); r.sambell@ecu.edu.au (R.S.); 6 Caring Futures Institute, Flinders University, Adelaide, SA 5001, Australia; Rebecca.golley@flinders.edu.au

**Keywords:** early childhood education and care, childcare, long day care, infant, toddler, preschool, nutrition, dietary guidelines

## Abstract

Early childhood is a critical stage for nutrition promotion, and childcare settings have the potential for wide-reaching impact on food intake. There are currently no Australian national guidelines for childcare food provision, and the comparability of existing guidelines across jurisdictions is unknown. This project aimed to map and compare childcare food provision guidelines and to explore perspectives amongst early childhood nutrition experts for alignment of jurisdictional childcare food provision guidelines with the Australian Dietary Guidelines (ADG). A desktop review was conducted and formed the basis of an online survey. A national convenience sample of childhood nutrition experts was surveyed. Existing guideline recommendations for food group serving quantities were similar across jurisdictions but contained many minor differences. Of the 49 survey respondents, most (84–100%) agreed with aligning food group provision recommendations to provide at least 50% of the recommended ADG serves for children. Most (94%) agreed that discretionary foods should be offered less than once per month or never. Jurisdictional childcare food provision guidelines do not currently align, raising challenges for national accreditation and the provision of support and resources for services across jurisdictions. Childhood nutrition experts support national alignment of food provision guidelines with the ADG.

## 1. Introduction

Early childhood (birth to 5 years) is a key life stage for implementing strategies to improve nutrition and prevent obesity [1,2]. Food preferences and dietary patterns are developed in early childhood and can influence later habits [3,4]. Nutrition in early childhood influences both short- and long-term health including growth and development, risk of overweight and obesity, and related chronic disease [2,5,6]. 

The number of infants and young children with overweight or obesity has increased globally from 32 million in 1990 to 41 million in 2016 [7], with greater prevalence and rate of increase in high-income compared to low- and middle-income countries [2]. Similar to adults, the key determinant of overweight and obesity in children is an energy-dense and nutrient-poor diet coupled with insufficient physical activity [2,7].

In Australia, almost one in four children aged 2–4 years have overweight or obesity [8]. Compared against Australian Dietary Guidelines (ADG) [9], only 20% of children aged 2–3 years eat sufficient vegetables, decreasing to 3% by school age [10]. Moreover, almost a third of total energy intake of 2–3 year olds is from discretionary foods (those high in kilojoules, saturated fat, added sugars and/or salt [9]), increasing to nearly 40% by the age of 4–8 years [10]. Similar dietary patterns are seen even before two years of age [11]. The ADG contains age-specific recommendations aiming to address these health and nutritional issues in the population [9]. There are five food groups: vegetables (including legumes and potatoes but not hot chips), fruit, grain (cereal) foods (mostly wholegrain and/or high cereal fibre varieties), meats/alternatives (including poultry, fish, eggs, tofu, nuts, seeds and legumes/beans but not processed meat) and dairy/alternatives. The standard measures for each of these food groups are termed “serves”, which reflect defined gram or kilojoule amounts across the different foods within the group. There is also an allowance for unsaturated spreads and oils, and discretionary choices.

Poor diet quality and high rates of childhood overweight and obesity in Australia and internationally indicate a need for early childhood interventions that focus on developing lifelong healthy eating habits and obesity prevention [2,12]. The family environment is traditionally recognised as the primary determinant for influencing children’s eating behaviours and food preferences [13]. However, societal changes in mothers’ workforce participation in developed countries [14] have resulted in childcare services sharing this role as a proxy-family environment [15].

Childcare services, also known as early childhood education and care (ECEC) or early care and education (ECE) services, are commonly accessed by young children, with two thirds of Australian children aged 1–4 years attending some form of childcare [16]. Of the available childcare options, long day care is utilised by the majority of families [17]. Long day care services are those which operate at least eight hours per weekday for children who are not yet attending school, with nearly 700,000 Australian children attending [17]. The greatest proportion of long day care attendees are 2–3 year olds (50% of Australian children of this age attend), followed by 4 year olds (38%) and 1 year olds (36%) [16]. On average, children attend long day care for 29 h per week and 19% attend for more than 40 h per week [17]. Another childcare option is family daycare, provided in-home, for more than 150,000 children nationally [17]. Children spend on average 27 h per week in family daycare services [17]. Childcare services in Australia provide children with a lunch and a mid-meal snack during both the morning and afternoon as a minimum. For most services, food is prepared and cooked on site from assembled ingredients by a cook or chef. Alternatively, parents might provide the food as packed lunchboxes. There is no data on the proportion of services using each method, but in a number of jurisdictions, most or all centres conduct all food preparation on site. Hence for some children, childcare food provision is a major contribution to their overall weekly food intake [18].

These childcare settings therefore provide a high level of exposure to food environments and feeding practices to a large number of children and for prolonged periods of time during a critical developmental stage. It is an ideal setting for nutrition promotion with extensive reach. The potential for childcare services as ideal settings for promoting healthy eating behaviours is recognised internationally [2,19]. Systematic reviews demonstrate that public health efforts and nutrition-related interventions directed at childcare can have positive impacts on children’s dietary intake and food choices [20]. Despite this, several studies have identified food provision as being of poor nutritional quality [18,21,22,23,24]; lacking important nutrients vital for optimal child growth and development [21]; not meeting recommended dietary requirements, especially meat and alternatives, dairy and vegetable consumption [21,24]; and regularly providing discretionary foods [22].

The use of policy is considered essential to create leverage within or between sectors and to provide a coordinated and coherent approach in the implementation of best practice guidelines and strategies [25]. The need for national childcare policies to guide food provision is recognised in other countries as well as in Australia [26,27]. The availability of a national policy lever for childcare services in Australia is a relatively recent occurrence. In 2012, the Australian Children’s Education and Care Quality Authority (ACECQA) was established as a national accreditation body for early childhood education and care services. ACECQA provides support across the eight Australian jurisdictions which undertake accreditation of services to assess that they meet all National Quality Standards. These standards stipulate “healthy eating … promoted and appropriate for each child” (Standard 2.1.3) [28], and concurrently, the Education and Care Services National Regulations require that services provide food which is nutritious; adequate in quantity; and takes into account each child’s growth, developmental, and cultural needs [29]. The Australian childcare sector is responding by shifting focus from jurisdictional guidelines to practice that is in line with the National Quality Standards. 

One example of best practice is the use of food provision guidelines, also known as menu planning guidelines, to support food prepared by childcare sites and provided to children while in care [30]. The intent of these guidelines is for food provision to be consistent with and age- and gender-specific ADG [9]. Implementation of food provision guidelines, either alone or as part of a multi-strategy intervention, have been shown to improve children’s dietary intake [18,31].

In Australia, despite the overarching National Quality Standards, there is no set of national childcare food provision recommendations to operationalise the National Quality Standards. Several jurisdiction-specific food-provision guideline resources, based on interpretations of the current 2013 ADG [9] and the nationally developed Australian Get Up and Grow [32] resources, are used as an adjunct to the nutrition-specific National Quality Standards (QA2, standard 2.1.3). To date, however, a systematic evaluation of existing jurisdictional resources has not been conducted. Therefore, differences between these resources and what it would take to achieve a nationally consistent approach to food provision in childcare is currently unknown. 

Jurisdictional differences may cause implementation challenges in providing nationally consistent healthy foods and drinks across childcare services. It also makes assessment of services against a national standard variable and possibly inconsistent. Hence, assessing the alignment of jurisdictional food provision guideline resources and determining the practicalities of harmonising these nationally are priorities to improve early childhood nutrition in Australia. 

The primary purpose of this study was to assess alignment of food provision guidelines between Australian jurisdictions and to seek expert opinion for national alignment based on the ADG.

The objectives of the study were the following:

1. to map and compare existing food provision guidelines for childcare services across the eight Australian jurisdictions and
2. to explore perspectives and to determine the level of agreement amongst early childhood nutrition experts for alignment of jurisdictional childcare food provision guidelines with the Australian Dietary Guidelines.

## 2. Materials and Methods

### 2.1. Study Design

This descriptive study involved two phases. Phase 1 was a desktop review to map the existing national and jurisdictional recommendations on food provision in childcare. Phase 2 was a survey of early childhood nutrition experts, with items informed by the desktop review. Phase 1 did not require ethics review. Phase 2 was approved by the ethics committee at Edith Cowan University (2019-00027) and Deakin University (2019-073).

### 2.2. Phase 1 Mapping of Jurisdictional Food Provision Guidelines for Childcare

The mapping phase of this project was conducted between December 2018–January 2019. A research assistant completed both data source searching and extraction, which was cross-referenced by an author for completeness and accuracy (P.L.).

#### 2.2.1. Data Source

(In Australia, there are eight major jurisdictions: six states (New South Wales, Victoria, Queensland, Western Australia, South Australia and Tasmania) and two Territories (Northern Territory and Australian Capital Territory). A list of national and jurisdictional government websites was developed based on the authors’ knowledge of relevant health and education policy, supplemented by emails to content experts including members of the National Nutrition Network–Early Childhood Education and Care (NNN–ECEC). The NNN–ECEC is an Australia-wide network of government and nongovernment practitioners who work directly with childcare centres as well as researchers with expertise in early childhood and childcare nutrition research, practice and policy. Members of the network are knowledge brokers and are considered food and nutrition content and practice experts for this sector. Members collaborate to identify and act upon priority nutrition issues and to facilitate a healthy food environment in ECEC settings through systemic change. 

Identified website content and embedded files were screened to identify policy, guidelines or recommendation documents. Documents included in the mapping exercise contained recommendations on the type and amount of food or drinks to be provided in childcare. While feeding practices in childcare are also known to be a relevant component of meals and to influence child intakes, we do not have national guidelines for feeding practices in childcare, and review of these was beyond scope for this paper. 

#### 2.2.2. Data Extraction

Data was extracted from website content, policy and guideline documents using a data extraction table developed for the purposes of this study. Data extracted included servings recommended for the five food groups (vegetables, fruit, grain (cereal) foods, meats/alternatives and dairy/alternatives) plus fats and oils, and discretionary choices; frequency and definition information for five food groups; and any additional recommendations regarding menus or food provision, e.g., infant feeding practices, guidance on vegetarian meals and use of full-fat dairy products. 

Similarities and differences across jurisdictional guidelines were identified by comparing jurisdictional source, age cohort, food group serves, meals/snacks and serving sizes/weights. Jurisdictional recommendations were compared to the ADG for 2–3 year olds [9]. The ADG age groups available for use as reference categories are 7–12 months, 13–23 months, 2–3 years or 4–8 years, with substantial differences between age groups for some food groups. The ADG for 2–3 year olds was used as the benchmark, as this age group has the highest childcare attendance rates (approximately 50% of children this age attend long day care) [16]. All measures were converted to standard serves and units according to the ADG (2013) for consistency and comparability. 

Phase 2 used graphical representations of current recommended serves for each jurisdiction and compared these against 50% of the respective ADG food group serves for 2–3-year-old children. Where the recommendation is a range (e.g., 1–2 serves) the minimum was used. The level of at least 50% of the ADG recommendation was used as a benchmark as research and historical guidelines cited this amount as an appropriate proportion of food/nutrient recommendations to be provided in a long day care setting for one main meal and two snacks [33,34,35]. These graphs were embedded in the online survey used in phase 2.

### 2.3. Phase 2 Surveying of Early Childhood Nutrition Experts

#### 2.3.1. Survey

The online survey was distributed utilising Qualtrics [36]. The information and consent letter formed the first part of the survey, and with consent, participants were able to progress. Initial descriptive questions included jurisdiction, membership of the NNN–ECEC, current place of employment and position title, role expertise and involvement in food provision policy development.

The survey stipulated a focus on the childcare settings of long day care and family day care, given that children usually spend full days and consume one main meal and two snacks in these settings as opposed to the shorter hours of occasional care. Using the Australian jurisdictional guidelines from phase 1, survey items were generated to translate these guidelines into quantitative questions with open-ended comments. 

For each of the five food groups, respondent agreement to food group provision recommendation with at least 50% of the ADG for 2–3 year olds was assessed using a graphical representation for each food group (see Supplementary Figure S1). An additional item for each food group asked whether providing that quantity would be realistic. Further questions explored participant views on benchmarking selections and serving size terminology: “Recommendations in the above questions for each core food group have been standardised for comparison to the ADG based on a reference child of 2–3 years old and the assumption that at least 50% of core foods should be provided. Do you agree with the assumption that at least 50% of core foods should be provided across morning tea, a midday meal and afternoon tea? Do you agree with the assumption to standardise food group recommendations based on the 2–3-year-old’s ADG?” and “Which serving size terminology do you think is most appropriate for use in ECEC food provision recommendations?”.

Based on phase 1, many guidelines made statements about *frequency* as well as *quantity* of providing food groups and foods within those groups; therefore, a further series of questions asked about recommended *frequency* for specific food and meal types. Twenty items asked respondents “*How frequently do you think the following foods should be served within an ECEC Centre?*” (items related to foods such as beef, vegetarian meals and cheese) and used a 7-point response scale (never, occasionally, ≤once a month, 1×/fortnight, 1×/week, 2×/week, 3×/week and every day). Participants could select more than one frequency to indicate a range, so the proportion of total responses as well as the proportion of respondents selecting any response were considered in the analysis. 

For discretionary foods, respondents were asked to report on the suggested frequency that these foods should be served utilising the same 7-point scale described above. One question asked about “overall discretionary items”, with seven sub-questions relating to specific discretionary foods which were mentioned in jurisdictional guidelines, including sugar sweetened beverages, sports/energy drinks, biscuits cakes and slices, lollies and chocolate bars, muesli bars, high-fat pastries and fried foods. (In Australia, “biscuits” and “slices” are forms of individual or sliced sweet baked items in individual portions; “lollies” refers to sugar-based confectionary; and “muesli bars” refers to sweetened bars usually made from oats, dried fruit and/or nuts and usually sold commercially in individual packages. Examples of “fried foods” given in the survey were hot chips and battered fish.) For analysis, responses from 1×/week to every day were combined due to few responses.

#### 2.3.2. Recruitment

The target group was experts in early childhood nutrition that were (a) members of the NNN–ECEC (eligible *n* = 29), and (b) non-NNN–ECEC members such as dietitians, nutritionists and researchers (unknown eligible population). As the primary focus of this work was related to nutritional requirements rather than setting practicalities and as there is minimal employment of nutritionists or dietitians directly in childcare settings, experience within this setting was not a criteria as it may have further limited the sample size. Initial recruitment was through a convenience sample of NNN–ECEC members who were invited to participate via email and encouraged to forward the invitation to other early childhood nutrition experts from within their own personal contact network for snowball sampling [37]. Members of this group were deemed research and practitioner experts for this sector. The survey was initially open for four weeks in March 2019. Due to small recruitment outside the NNN–ECEC, further recruitment strategies were employed to advertise the survey for an additional four weeks in August 2019, through circulation to professional bodies and networks relevant to early childhood nutrition: Dietitians Association of Australia (Deakin, Australian Capital Territory), Dietitian Connection (Mt Gravatt Queensland), Nutrition Society of Australia (Crows Nest, New South Wales), Health Promotion Network from NSW Office of Preventive Health (Liverpool BC, New South Wales) and registered attendees for a childcare-focused conference workshop. Inclusion criteria in the advertisement and survey consent process asked participants to self-identify as “early childhood nutrition experts”.

#### 2.3.3. Consensus

Consensus is sought where there is a discrepancy in interpretation and parties seek to reach agreement on a course of action [38]. Consensus methods are used to develop guidelines including appropriate indications for interventions [39], and consensus building is considered an important component of participatory planning [40]. To establish levels of agreement with statements relating to jurisdictional recommendations, purposive sampling was undertaken and included individuals who were selected based on their membership to the NNN–ECEC and were self-reported experts in childhood nutrition. Consensus was defined as having ≥80% agreement between respondents. In previous studies within the nutrition and health sector, a minimum of 80% agreement to reach consensus has been required. This includes projects establishing an operational definition of Sarcopenia in Australia and New Zealand [41] and for definition statements for frailty [42,43]. 

#### 2.3.4. Analysis

Quantitative responses were collated, and the number and proportion of respondents responding to each survey question were reported. Descriptive statistical analysis was conducted using Stata, version 15 (Statacorp, College Station, TX, USA). Data from open-ended questions were analysed to identify key points, issues and themes raised by respondents. Co-authors (A.W. and A.S.) themed the open-ended questions, and these were reviewed independently by two separate authors (R.S. and A.D.) with any differences in theme coding discussed and adjustments made.

## 3. Results

### 3.1. Phase 1 Mapping of Jurisdictional Food Provision Guidelines for Childcare

The childcare food provision guidelines used by the eight Australian jurisdictions are presented in Table 1. Western Australia (WA) had no specific food provision guidelines, with services generally referring to the *Supporting Nutrition for Australian Childcare* (SNAC) website, where information is based on the *Start Right Eat Right* resources and the Australian Dietary Guidelines [9]. South Australia (SA) had no current government-endorsed guidelines but is supported by Nutrition Australia Victoria with training and menu planning resources. The *Start Right Eat Right* award program which included menu planning guidelines was active and widely implemented in SA between 2001–2013 and is still utilised by SA long day care services [44]. Information for the 2–3-year-old age group was tabulated, with two jurisdictions’ guidelines specific to this age group and other jurisdictions’ guidelines for a wider age range.

#### 3.1.1. Quantity of Food Provision

A summary of the recommendations regarding the five food groups, fats and oils, and discretionary items as well as a comparison to 50% of ADG [9] are shown in Table 1. Six jurisdictions used serve sizes consistent with the ADG, while Victoria (VIC) and South Australia (SA) used “child” serve sizes for some food groups, which were similar to but not entirely consistent with 50% of the ADG. 

Jurisdictional guidelines differed in their alignment of providing at least 50% of the ADG recommended serves for a 2–3 year old. Grain (cereal) foods were the only consistent recommendation across all jurisdictions, aligning with 50% ADG serves for a 2–3 year old. There were marked differences for vegetable recommendations; three jurisdictions recommended a minimum amount less than 50% of the ADG for 2–3 year olds (SA, VIC and Tasmania (TAS)), while two recommended more than 50% of the ADG for 2–3 year olds (New South Wales (NSW) and Northern Territory (NT)). Regarding discretionary foods, three jurisdictions recommended that these foods should not be included or be on the regular menu, three allowed amounts up to once per week or once per day, and one did not provide discretionary stipulations. The NT, Australian Capital Territory (ACT) and Queensland (QLD) were the jurisdictions which provided age-specific recommendations which separated 2–3 year olds from older children. 

#### 3.1.2. Quality of Food Provision

In addition to recommendations regarding food quantity, an additional 152 parameters regarding dietary quality, variety and frequency were identified across the eight jurisdictions. These included items such as type of milk to be served, use of wholegrains, frequency of iron rich foods, and some guidelines specifying weekly or fortnightly frequency for specific food types (e.g., beef, lamb, kangaroo, bacon, dried fruit, yoghurt and cheese). Guidelines in NSW were the most stringent on iron intake, requiring the highest frequency of iron rich foods per day of all the jurisdictions. Guidelines in VIC, NSW, NT and TAS all emphasised variety across the menu. An example of jurisdictional differences is illustrated in relation to the food group “lean meats and poultry, fish, eggs, tofu, nuts and seeds, and legumes/beans” [47], with some jurisdictions having separate recommendations for red meat from other meats/alternatives [35,45] and some further specifying white meat, fish and vegetarian meals [50]. A full list of these guidelines is provided in Supplementary Table S1.

### 3.2. Phase 2 Surveying of Early Childhood Nutrition Experts

#### 3.2.1. Participant Characteristics

Forty-nine valid responses were received, after excluding one survey from a country other than Australia, one duplicate survey (initial one retained) and one ineligible participant (not a childhood nutrition expert). The largest number of respondents were located in NSW (31%, *n* = 15), with respondents from seven jurisdictions excluding Tasmania. Respondents identified as dietitians (71%), registered nutritionists (10%) or other (18%, including health promotion officers, childcare educators, childcare assessors, nutritionists still applying for registration and academics). There were 20 NNN–ECEC members and 29 additional non-NNN–ECEC childhood nutrition experts. When considering childcare food provision policy, the majority (61%) had no involvement in policy development, 24% had current involvement and 18% had previous experience (Table 2).

#### 3.2.2. Views on Food Provision Aligning with at Least 50% of ADG Serves for 2–3-Year-Old Age Group

Most respondents (92%, *n* = 45) agreed that at least 50% of the ADG five food groups’ serve recommendations should be provided across morning tea, a midday meal and afternoon tea in childcare services. Of the 43 comments on this item, the most common (26%, *n* = 11) related to this was a minimum requirement. The other most frequent comments questioned how this should be applied or operationalised in services which provided more meals (breakfast or late snack) or fewer meals (main meal is brought from home) (*n* = 4) and were about the evidence and modelling behind applying this percentage (*n* = 3). Of the four respondents who disagreed, two suggested that more than 50% should be provided, one respondent suggested less be provided (due to considerations of waste and not promoting excess energy intake), and another suggested considering more options and evidence. 

For the item assessing choice of the 2–3-year-old age group for standardising food group recommendations, two thirds of respondents agreed (67%; *n* = 33, with one non-responder). The most common comments provided by those who disagreed (*n* = 15) included that further investigation or more information was needed, such as proportion of children in each age range, and that specific recommendations for each age group should be considered. 

#### 3.2.3. Views on Five Food Group Recommendations Aligning with 50% of ADG for the 2–3-Year-Old Age Group

**Figure 1:**
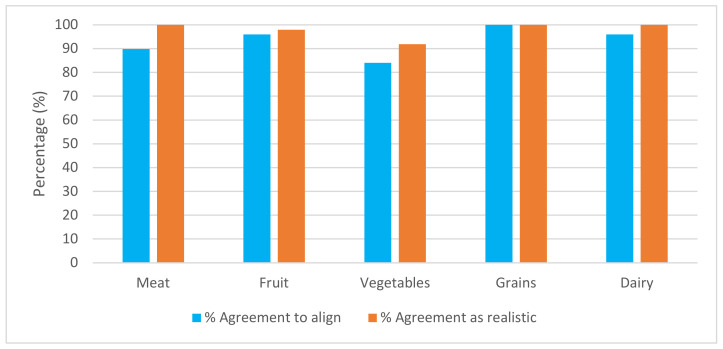
Respondents’ views on whether jurisdictional recommendations should align with 50% of ADG for each food group and whether this is realistic.

When assessed by individual food groups, there was good agreement (≥80%) for each of the five food groups to align with 50% of ADG recommendations (Figure 1); hence, consensus was reached. There was greatest agreement for the grain (cereal) (100%), fruit and dairy (96% each) food groups. 

Further exploration of comments in relation to the two food groups with least agreement, vegetables (84%) and meat (90%), was undertaken. Eight respondents disagreed with the alignment of vegetables to the 50% ADG recommendation (equivalent to 1.25 serves), with six respondents preferring a greater amount. Reasons given (*n* = 6) were mostly related to inadequacy of vegetables in Australian children’s diets and utilising childcare as an opportunity to provide more. A main concern was that providing sufficient vegetables was a practical challenge for services. 

With regard to meat and meat alternatives, five respondents disagreed and preferred a greater number of serves. Three comments were related to insufficiency of intake of this food group at home, suggesting that centres should provide more iron-rich foods.

Respondents were also asked whether they felt that providing 50% of the ADG daily for each food group was realistic for a childcare service. There was ≥80% agreement that it would be realistic for services to do so for all ADG food groups, indicating consensus (Figure 1).

#### 3.2.4. Serves: Terminology and Size

Views on the most appropriate serve size terminology varied. “ADG serve sizes” were preferred by 76% (*n* = 37), with supporting comments related to standardisation, consistency and minimising confusion through alignment with current ADG serve size terminology. “Child serve sizes” were preferred by 16% (*n* = 8), as terminology was considered “simple” and more realistic to a child’s consumption and relatable for childcare service staff. Selection of “Other” (8%, *n* = 4) was followed by comments about a need to consider childcare service staff perspectives as well as consistency and clarity in any conversions from ADG serves and that use of both terms for different purposes might be appropriate.

The practicality of fractional serve sizes was also highlighted as an issue in various comments throughout the survey, such as for vegetables serves where 50% of ADG equates to 1.25 serves or approximately 95 g of vegetables. There was a suggestion that serve sizes could be simplified or rounded, for example, to match standard cup sizes (1 cup or ½ cup) to support translation into practice. 

#### 3.2.5. Recommended Frequency of Specific Food Types

Responses to the items assessing frequency of food provision showed consensus that fresh fruit, vegetables, whole grains and dairy foods should be offered daily, while fruit juice should “never” be offered (Table 3). There was greater variation in responses regarding how frequently different types of meat and vegetarian meals should be provided with most respondents choosing once per fortnight, or once or twice per week. Additional comments were related to specific meat and processed meat guidelines, made by 18 respondents, with the majority highlighting the challenges and impracticalities associated with such specifications and the need to be realistic and practical, to use discretion and moderation, and to consider cultural preferences and local food availability. Five respondents suggested meats be grouped as “red meat” (beef, lamb and kangaroo), “white meat” (chicken and pork) and “processed meat” (lean ham, bacon and sausages), with “red meat” only served once or twice a week. Consideration of environmental sustainability when making recommendations regarding meat provision was highlighted. 

#### 3.2.6. Provision of Discretionary Items

**Figure 2:**
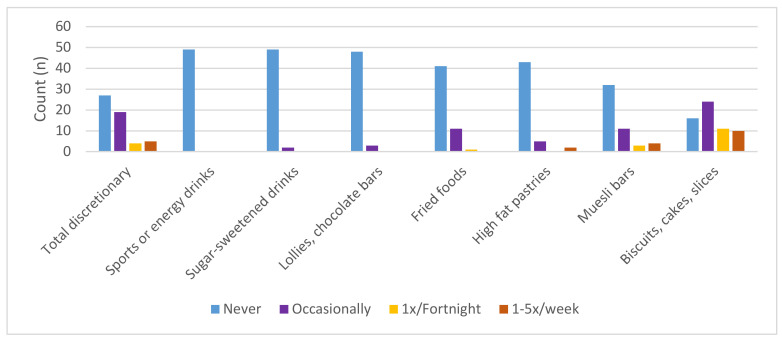
Number of respondents selecting each frequency to provide discretionary food and drink items: Responses for 1–5 times per week were combined due to few responses.

Most responses 94% (*n* = 46) indicated that discretionary items, overall, should be offered “never” or “occasionally: ≤once per month” (Figure 2). When types of discretionary foods were assessed separately, almost all participants agreed with never offering sports/energy drinks, sugar sweetened drinks and lollies/chocolate bars, whereas there was higher agreement that biscuits, cakes and slices could be offered fortnightly/weekly (39%) (Figure 2).

Forty respondents provided qualitative comments about discretionary items. The most common theme (*n* = 16) related to preparing or providing healthier versions:

“*Cakes or muffins may contain fruit/vegetable and be made low added sugar or fat*.”

Another common theme (*n* = 15) supported use for special occasions, celebrations and events, and views were conveyed that, “*Limited choices and quantities (sic) ok for occasional celebrations (e.g., quarterly) … not regular part of menu*”.

A third theme (*n* = 9) identified enough use of discretionary items at home or outside the childcare environment, with unhealthy diets leading to health problems. For example, a respondent highlighted that, “*Literature shows considerable environmental exposure to discretionary foods. (Long day care) need not be part of this regular exposure.*”

There was a diversity of comments relating to education, knowledge and difficulties in decision making regarding discretionary foods for policy makers, centres, staff and parents (*n* = 8), with comments such as, “*It is very challenging for settings to have the capacity, knowledge and tools to do this.*”

With this educative aspect in mind, a few respondents noted that discretionary items were good for cooking activities (*n* = 5) and included opinions:

“*Biscuits, cakes and slices are good options to learn to cook for young children in this type of setting*.”

Others regarded discretionary food use as an opportunity to teach children about these foods within a healthy diet (*n* = 6) and stated that these foods “*can provide a good opportunity to teach children about occasional/sometimes and everyday foods*”.

## 4. Discussion

This descriptive study is the first to scope the guidelines used across Australia to understand the jurisdictional differences of food provision in childcare services. The survey of childhood nutrition experts within academia and practice is the start of a conversation about how guidelines could be harmonised nationally. The aim of this study was to determine alignment and agreement between Australian jurisdictional childcare food provision guidelines, with exploration of early childhood nutrition experts’ perspectives on alignment with the ADG. Expert surveys to gain opinions and consensus have been conducted in other fields [41,42,43] but not on the topic of childcare nutrition. Other studies have compared childcare food provision or menus to jurisdictional or national guidelines [18,21,24,52,53], and one US study compared state regulations with national standards but focussed more on menu documentation than food quality [54]. Further related examples are a US study which collectively considered jurisdictional guidelines compared with a national benchmark on cultural and religious food incorporation [55] and another which compared US jurisdictional infant feeding regulations [56]. However, no other studies in Australia or elsewhere are known to have compared food provision quantity guidelines with each other or to have assessed alignment of childhood nutrition expert opinions with those guidelines. 

Variations in jurisdictional guidelines were identified in this study. While all were based on the ADG [9,57], it seems that different parameters and assumptions were used in interpreting and translating the ADG, giving rise to observed jurisdictional guideline differences. The majority of jurisdictional guidelines do not provide a clear rationale as to how the ADG for different age groups were translated into specific childcare food provision guidelines, what assumptions were made about the nominal (50%) daily requirement and reference age categories, or other factors such as rounding which occurred. Some of the guidelines also incorporated ranges, without stipulating how these were to be used. Furthermore, there was variability in how much information was publicly available and identifiable in online searches versus upon request.

The survey component of this study explored the details of constituent components of food provision guidelines, particularly alignment to at least 50% of ADG, using a reference age group to simplify the practicalities of menu planning. A minimum of 50% of daily food provision while in care has been suggested over the last 20 years. During this time, recommendations have varied between 50–75% and have aligned with nutrient recommended dietary intakes for key nutrients [33,58,59,60,61] instead of food groups [21]. More recently, studies have made comparisons to food groups to adapt to the changing dietary guidelines [21,24] and jurisdictional government recommendations [35]. This study found that more than 80% of childhood nutrition experts were in agreement that food provision guidelines should align with the ADG quantities of the five food groups, that this was a realistic goal for services and that a minimum of 50% of food groups should be provided whilst a child is in care.

This study showed that less than 80% of childhood nutrition experts were in agreement regarding the use of the 2–3-year-old age group as a reference age group for food provision within childcare services. While this age group has the highest proportion of children attending childcare, this study revealed important considerations related to nutritional adequacy and food wastage if just one age group is deemed the reference and if different services have different proportions of child age groups. These findings lead to the question of whether it would be more appropriate to have different food provision guidelines for different age groups in alignment with the ADG, for example, <2 years, 2–3 years and 4–5 years. Currently, ACT, QLD and NT have age-specific recommendations separating children three years and younger from those four years and older, while NSW separates those under two years [35,45,46,47]. Other researchers have also suggested benefits of separate feeding policies for the infant age group [62]. Menus could potentially reflect specific provision by ADG age group segmentation if online menu planning tools and health professional software algorithms were adjusted to accommodate these; however, the practicality for services to work with and operationalise such requirements would need to be investigated.

Serve size terminology across jurisdictions was predominantly in line with ADG serves, though two jurisdictions used “child” serve sizes for some food groups. While most survey respondents agreed with the use of the ADG standard serve, a number of comments highlighted the need for consideration of practicality for childcare service staff. Victorian food provision guidelines currently use “child” serves, an adaptation from the ADG standard serves, to provide practical recommendations for services to implement easily [9,50]. This allows consideration of practicalities in food service, for example, where young children eat smaller quantities than ADG serves, or in line with comments in this study where some ADG standard serves equate to fractions which can present challenges for practical translation at the service level. Ultimately, consistency and consultation with the sector are likely to be key to determining if utilising a standard serve would impact food group provision.

Discretionary food recommendations differed between jurisdictions more than other food types, ranging from not being included on the regular menu to an allowance of once per day. However, there was a high level of agreement between study respondents that discretionary foods should never or rarely be provided, with acknowledgement that children frequently overconsume these foods, aligning with existing Australian consumption data [10,11]. While the ADG has an allowance of up to one serve daily of discretionary foods for 2–3 year olds [9], additional information on the ADG website clarifies and aligns with respondents views that “Childcare (foods should) not include discretionary foods and drinks; (these) should be kept for special occasions.” [51]. While many respondents said biscuits, cakes and slices could be offered more often, it was frequently commented that healthier versions of these should be served, and only three respondents agreed with offering discretionary foods more than once per month. Comments about the occasional use of discretionary foods for celebrations or the value of inclusion from an educative viewpoint suggest a need for further investigation of such perspectives. The inclusion of fats and oils in some guidelines as discretionary items and challenges with interpreting and extrapolating the ADG for these items also add complication for this food group.

As well as differences in food group quantity recommendations, guideline differences regarding dietary quality, variety and frequency were identified across the eight jurisdictions. These differences made translation and adoption of a nationally consistent approach challenging. Previous research has found that childcare staff commonly rely on personal knowledge and that some services do not use evidence-based guidelines such as the ADG when determining nutritional adequacy of the food provided to children [62,63]. Moreover, poor awareness of jurisdictional guidelines and evidence-based recommendations have been identified at the service level. The benefits of alignment would be an increase in service awareness of regulatory requirements and a reduction in nutrition misconceptions that could negatively impact the nutrition environment [63]. Jurisdictional policy alignment could also reduce repeated mistakes and unnecessary duplication of research [64] and could ensure that recommendations consistently adhere to the best available evidence.

The National Quality Framework (NQF) provides a national approach to regulation, assessment and quality improvement for childcare across Australia. National alignment, through harmonisation of food provision guidelines, would support consistency in education, monitoring and assessment for the childcare sector. For ACECQA, national alignment would make assessments of the nutrition component of National Quality Standard 2.1.3 more consistent and would streamline assessor training as well as nutrition sector support to this regulatory body. For services, there would be clearer and consistent guidance on meeting National Quality Standard requirements. For training and educational bodies, this would foster consistency and quality of messaging in addition to shared resourcing across the jurisdictions.

The differing jurisdictional recommendations have resulted in a number of similar guidelines being used and resources and training being developed to support these, to a variable extent, within each jurisdiction. National alignment could improve service support by allowing consistent and shared national training and resources [62], by reducing unnecessary duplication and by allowing new developments to benefit the entire sector. For most jurisdictions, a harmonised approach could result in quite minimal changes to their existing guidelines, as many jurisdictions are currently close to alignment. It is therefore recommended that movement towards a national harmonised approach should build upon rather than duplicate existing resources. Such collaboration may also offer opportunities to address emerging food provision challenges including changing meal-time environments, parent work and childcare hours, sustainability, allergies, cultural diversity and food security, which are not unique to each jurisdiction.

A study of 1173 early education and care programs across 10 states of the US, from 2012–2017, found that a multisector approach to promoting policies and practices related to child nutrition could lead to improved food environments for young children in childcare settings [65]. They developed consistent, centralised supporting materials and allowed for tailoring in the approach by individual services, similar to our proposal of harmonisation. That study demonstrated that collaborative partnerships led to broad implementation of best practices in relation to child nutrition and that this model was found to be complementary rather than duplicative to existing programs and initiatives [65]. 

Limitations of this study relate to the unknown number of childhood nutrition experts; hence, it is unknown what proportion of eligible participants is represented by our sample of 49 or whether our sample reflects the geographical spread of eligible participants. Face and content validation were undertaken with nutrition and industry experts, but survey piloting, repeatability and validity-testing were limited due to the short time frame and limited sample. Due to the small available sample, most authors participated in the survey to ensure that the full views of the NNN–ECEC were represented. 

As identified by some participants in this survey, further understanding of childcare food wastage and related strategies is also needed, in line with increased focus on environmental considerations in the broader health and nutrition context [66,67]. Further investigation is also required into the consequences of harmonised guidelines for services utilising lunchbox food provision from home and/or where attendance hours vary and might include additional food provision (e.g., breakfast) or only a few hours of the day (e.g., occasional care). Additionally, investigation into guidelines and policies regarding feeding practices and mealtime environments beyond the foods served was outside the scope of this study but is important given these also influence child food intakes. 

Seeking views and collaborations from the childcare sector (e.g., childcare services and service staff and peak bodies) will be an important next step, particularly in determining how to operationalise a harmonised approach to food provision guidelines within services. Legitimising and empowering childcare service staff regarding their potential to influence food provision and the feeding of young children in their care requires clear policies and guidelines [68,69]. Successful implementation of guidelines is dependent on stakeholder engagement and understanding of the rationale to change [70,71,72]. 

## 5. Conclusions

As a first step towards achieving nationally consistent food provision guidelines for Australian childcare services, this study provides a comprehensive understanding of current jurisdictional food provision recommendations and views on these by early childhood nutrition experts. This study indicates that food group recommendations should align with the ADG and that this is a realistic recommendation to implement in childcare services. Implementing consistent nutrition guidelines for childcare services across each Australian jurisdiction would require relatively little modification to most existing guidelines and could positively influence translation of food provision guidelines for resource development, service implementation and sector accreditation. Given the well-recognised importance of optimal nutrition in early childhood and the high rates of childcare usage in Australia, this is a strategic pathway for nutrition promotion to contribute to nationwide efforts of obesity prevention and to improve the lifelong health outcomes for children. 

## Figures and Tables

**Table 1:** Mapping of existing jurisdictional food provision guidelines for childcare against provision of 50% of the 2013 Australian Dietary Guidelines (ADG) recommendations [9] for 2–3 year olds.

Jurisdiction ^a^	50% of ADG (2013)	ACT	NSW	NT	QLD	SA (and WA-SNAC)	TAS	VIC
**Source**		Nutrition Australia, ACT Government; 2016 [45]	Caring for Children, Ministry of Health NSW; 2014 [35]	NT Government; 2016 [46]	Nutrition Australia Qld, Food Foundations; 2018 [47]	Start Right, Eat Right, DHHS; 2012 [48]	Move Well, Eat Well, DHHS; 2016 [49]	Nutrition Australia Vic; 2012 [50]
**Age group covered by recommendations**	2–3 years	2–3 years ^b^	2–5 years	≤3 years ^b^	2–3 years ^b^	>6 months	1–5 years	1–5 years
**Daily intake proportion to be provided in care**		50%, not further specified	“at least 50% of the recommended dietary intakes (RDI) for all nutrients”	No information provided	“at least 50% of ADG core food group requirements”	“at least 50% of daily nutritional requirements”, “50% of the serve recommendations of the AGHE”	“half their daily nutrition requirements”	50%, not further specified
**Meals/snacks**		1 main meal + 2 mid-meals	1 main meal + 2 mid-meals	1 main meal + 2 mid-meals	1 main meal + 2 mid-meals	1 main meal + 2 mid-meals	1 main meal + 2 mid-meals	1 main meal + 2 mid-meals
**Serve sizes**		ADG	ADG	ADG	ADG	ADG—fruit; veg; grains Child ^c^—dairy; meat	ADG	ADG—veg; grains Child ^c^—dairy; meat; fruit
**Vegetables/legumes**	1.25 serves = 95 g of cooked vegetables	1.25 = 95 g cooked	2 = 150 g cooked	2 = 150 g	1.25 = 95 g cooked	1 = 75 g cooked	1 to 2 = 75–150 g fresh/cooked	1 to 1.5 = 75–110 g fresh/cooked
**Fruit**	0.5 serves = 75 g fresh fruit	0.5 = 75 g apple	1 = 150 g apple	1 = 150 g	0.5 = 75 g apple	0.5 = 75 g apple	0.5 = 75 g apple	1 child serve ^c^ (0.5 ADG) = 75 g fresh/canned/cooked
**Grain (cereal) foods**	2 serves = 2 slices of bread; 150 g cooked rice/pasta/noodles	2 = 2 slices bread	2 = 2 slices bread	2 = 2 slices bread	2 = 2 slices bread	2 = 2 slices bread	2 = 2 slices bread	2 = 2 slices bread
**Meats/alternatives**	0.5 serves = 50 g raw or 33 g cooked lean meat or 1 large egg	0.5 = 33 g cooked lean meat	0.75 = 75 g raw beef	0.5 = 50 g raw beef	0.5 = 50 g raw beef	1 child serve ^c^ (0.5 ADG) = 50 g raw beef	0.5 = 50 g raw beef	1 child serve ^c^ (0.5 ADG) = 50 g raw beef
**Dairy/alternatives**	0.75 serves = 185 mL milk; 30 g hard cheese	0.75 = 185 mL milk	1 = 250 mL milk	1 = 250 mL milk	0.75 = 185 mL milk	2 child serves ^c^ = 200 mL milk; 30 g cheese	1 = 250 mL milk	2 child serves ^c^ = 200 mL milk; 30 g hard cheese
**Fats/oils**	0.25 serves = 2–2.5 g/day unsaturated spreads/oils	No information provided	Classified as discretionary	Classified as discretionary	No information provided	Maximum 7 g/day	No information provided	Classified as discretionary
**Discretionary ^d^**	0 serves ^f^	No information provided ^e^	Should not be included on service menu ^e^	Not to be consumed on a daily basis	≤1/day	≤2/fortnight, for flavouring	Should not be offered on the menu ^e^	Should not be included in daily menu ^e^

^a^ Australian Capital Territory (ACT), New South Wales (NSW), Northern Territory (NT), Queensland (QLD), South Australia (SA), Western Australia (WA), Supporting Nutrition for Australian Childcare (SNAC), Tasmania (TAS) and Victoria (VIC). ^b^ ACT, NT and QLD include age-specific recommendations, with separate recommendations for some or all food groups for children 4 years and older. Recommendations for the 2–3-year-old age group are included here. ^c^ Child serve = used in some jurisdictions for fruit, dairy and meat/meat alternatives, usually similar to half ADG serve. ^d^ Foods high in kilojoules, saturated fat, added sugars and/or salt [9], which includes fats/oils in some jurisdictions. ^e^ The NSW guidelines also say “Milo^TM^ is a good source of iron; however, it should not be served every day.” (Milo is a fortified chocolate and malt powder) and “Sausages may be included on the menu from time to time (no more than once every two weeks)”. ACT guidelines also allow ham. VIC guidelines also allow lean ham or bacon × 1–2/week. TAS guidelines also allow lean ham or bacon × 1–2/week and lean sausages × 1/month. ^f^ While there is an allowance for discretionary choices in the ADG (0–1 serves per day), the associated Eat for Health Educator Guide also states “For younger children, up to about 8 years of age, discretionary choices are best avoided or limited to no more than ½ serve a day unless the child is taller or more active”. Furthermore, the ADG website states “Childcare (foods should) not include discretionary foods and drinks, … (these) should be kept for special occasions.” [51].

**Table 2:** Participant characteristics.

Characteristic	%	*n* (of 49)
Jurisdiction		
Australian Capital Territory	6%	3
New South Wales	31%	15
Northern Territory	4%	2
Queensland	14%	7
South Australia	10%	5
Tasmania	0%	0
Victoria	26%	13
Western Australia	8%	4
Profession		
Dietitian	71%	35
Registered Nutritionist	10%	5
Other	18%	9
Currently involved in policy	24%	12
Previously involved in policy	18%	9
Not involved in policy	61%	30

**Table 3:** Suggested frequency of provision of food items in childcare services: count of respondents ^a^ who agreed with each frequency option (*n* = 49 ^b^).

Food Item	Never	Occasionally	Once per Fortnight	Once per Week	Twice per Week	Three Times per Week	Every Day
Beef	2	3	13	**32**	24	5	1
Lamb	2	6	26	**30**	11	3	1
Kangaroo	5	**25**	17	18	8	2	1
Pork	3	11	25	**26**	10	2	1
Chicken	2	2	12	**30**	26	6	2
Fish	2	2	12	**34**	20	4	2
Lean ham	15	**21**	13	7	3	2	1
Lean bacon	21	**27**	7	4	1	1	1
Lean sausages	21	**26**	7	1	1	1	1
Vegetarian meals	2	3	8	**27**	22	12	7
Nuts	**21**	7	7	9	9	8	14
High fibre grains/cereals	0	0	0	0	0	1	**49**
Vegetables	0	0	0	0	0	0	**49**
Fresh fruit	0	0	0	0	0	0	**49**
Dried fruit	5	15	10	**20**	14	3	1
Fruit Juice	**41**	7	0	2	2	1	0
Unflavoured milk	0	0	0	1	2	6	**47**
Flavoured milk	**35**	11	1	4	2	2	2
Yoghurt	0	1	2	5	7	25	**31**
Cheese	0	1	2	3	10	27	**31**

^a^ The most commonly selected frequency for each item is in bold. ^b^ Multiple selections could be made for each food item to indicate that the respondent agreed with more than one option; hence, the number of responses for each item may be greater than 49.

## Supplementary Materials

The following are available online at https://www.mdpi.com/1660-4601/17/18/6793/s1, Table S1: Additional LDC menu planning guidelines identified across Australian jurisdictions, Figure S1: Example graphical representation: Jurisdictional serve recommendations for Vegetables in LDCs for 2–3 year olds (1 1/4 serves = 50% ADG).
