# Supplementary material for: Childcare Food Provision Recommendations Vary across Australia: Jurisdictional Comparison and Nutrition Expert Perspectives
Source: Int J Environ Res Public Health. 2020 Sep 17;17(18):6793. doi: 10.3390/ijerph17186793 (PMC7558074; doi:10.3390/ijerph17186793)
Supplement: Supplementary file 1 [file ijerph-17-06793-s001.pdf]

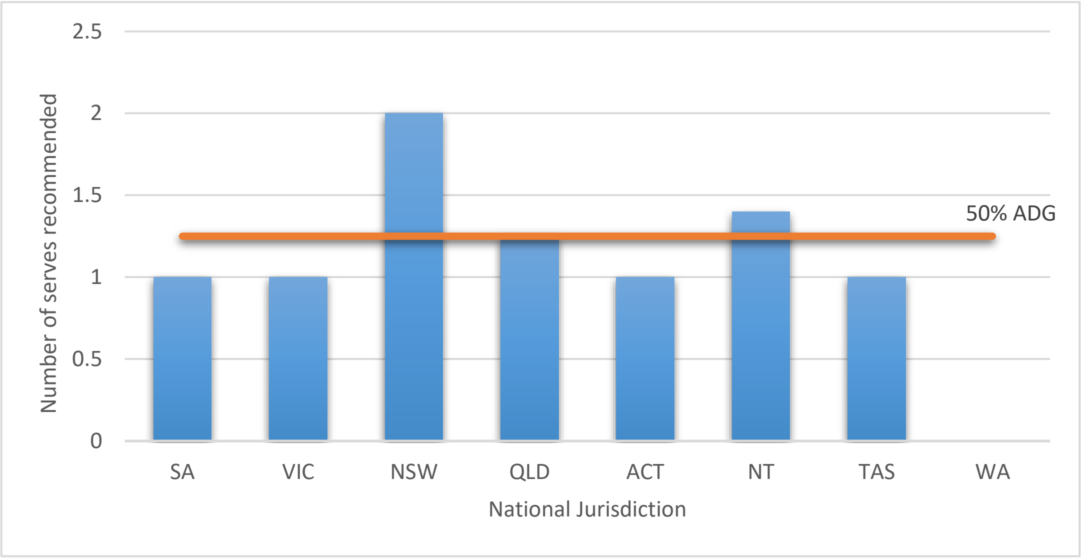

Supplementary Figure 1. Example graphical representation: Jurisdictional serve recommendations for Vegetables in LDCs for 2-3 year olds (1 1/4 serves = 50% ADG)

Supplementary Table 1. Additional LDC menu planning guidelines identified across Australian jurisdictions

	ACT	NSW	NT	QLD	SRER - SA	TAS	VIC (Healthy Eating Advisory Service)
**Meat/alternatives**						Lean ham or bacon <u>may</u> be included on menu once or twice per week *(either once as a major ingredient and once as a minor ingredient, or twice as a minor ingredient)*<br><br>Lean sausages may be included on the menu no more than once each month.<br>Lean ham, bacon and sausages are not offered on the menu on the same day.	Lean ham or bacon <u>may</u> be included on menu once or twice per week *(either once as a major ingredient and once as a minor ingredient, or twice as a minor ingredient)*
Red meat: beef, lamb, kangaroo	…included on the menu at least 3 times per week	.. included on the menu at least 6 times per fortnight<br><br>On each day that a red meat meal is served, a least 1 other iron-containing food is included on the menu e.g. wholemeal bread, breakfast cereal, dried fruit, milo	Include lean red meat on the menu twice a week		.. included on the menu at least 4 times per fortnight (1 serve = 50g raw)	…included on the menu at least 4 times each fortnight	.. included on the menu at least 4 times per fortnight
Pork, poultry, fish	White meat (skinless chicken, fish and pork) and non-meat meals are included on the menu at least 2 times per week	A variety of lean white (<u>chicken/fish/ pork/veal</u>) /non-meat meals are included on the menu up to 4 times per fortnight	Include lean white meat on the menu 3 times a fortnight		<u>Pork</u> or white meat: <u>chicken/fish/veal</u><br>Lean white meat is included on the menu at least 3 times per fortnight	Lean pork or poultry is included on the menu at least 2 times each fortnight.<br><br>Fish is included on the menu 1-2 times each fortnight	Lean <u>pork or poultry</u> is included on the menu at least 2 times per fortnight<br>Lean ham or bacon is allowed up to twice a week as a minor ingredient or once as a major ingredient<br><u>Fish</u> is included on the menu 1- times per fortnight

		On each day that a white meat meal is servec at least 2 other iron-containing foods are included on the menu e.g wholemeal bread, breakfast cereal, dried fruit, milo	Iron containing foods to be included when serving a white meat or vegetarian dish, such as wholemeal bread, spinach, broccoli, cauliflower, beans such as baked beans and lentils.		Served with at least 1 other iron containing food such as …. Spinach, legumes (baked beans and lentils), wholemeal bread, broccoli, spinach, cauliflower, cabbage		
Vegetarian meals	White meat (skinless chicken, fish and pork) and non-meat meals are included on the menu at least 2 times per week	A variety of lean white/non-meat meals are included on the menu up to 4 times per fortnight	Include a vegetarian meal on the menu at least once week		Included at least twice per fortnight	…included on the menu at least once each fortnight for variety	Included at least once per fortnight
	Non-meat meals are based on eggs, tofu or lentils/legumes	Non-meat meals are basec on eggs, cheese, tofu or legumes			Based on at least one of the following protein rich foods: eggs, cheese, milk, soy products (soy beans, tofu, legumes)	Include a food containing protein such as eggs, legumes (eg chickpeas), milk, yoghurt, cheese, soy products (eg tofu) or nuts (if centre policy allows).	Include protein such as eggs, legumes, milk, yoghurt, cheese, soy products (e.g. tofu) or nuts (if centre policy allows)
		On each day that a non-meat meal is served, at least 2 other iron-containing foods are included on the menu e.g wholemeal bread, breakfast cereal, dried fruit, milo	Iron containing foods to b included when serving a white meat or vegetarian dish, such as wholemeal bread, spinach, broccoli, cauliflower, beans such as baked beans and lentils		Served with at least 1 other iron containing food such as …. Spinach, legumes (baked beans and lentils), wholemeal bread, broccoli, spinach, cauliflower, cabbage	Vegetarian meals include a food containing iron such as spinach, legumes (eg chickpeas), baked beans, peas, tofu, eggs and broccoli	Served with an iron containing food such as …. Spinach, legumes, baked beans, tofu, eggs and broccoli
	Raw vegetables or fruit high in Vitamin C are served with the non-meat meal eg. Citrus fruit, tomato, broccoli, cauliflower, kiwifruit, capsicum, rockmelon.	Raw vegetables or fruit high in Vitamin C are served with non-meat meal e.g. citrus fruit, tomato, broccoli, cauliflower, capsicum, kiwifruit, rockmelon	Vitamin C rich foods to be included with vegetarian meals. Eg. Capsicum, broccoli, tomato, cabbage, peas, citrus fruit, kiwifrui rockmelon, strawberries.		A fruit or veg high in Vit C serves with a vegetarian meal: capsicum, broccoli, kiwi, cauli, red cabbage, citrus fruit, strawberries, rockmelon, peas, sweet potato, pineapple, tomato, zucchini	Vegetarian meals include a fruit or vegetable high in Vitamin C eg. Capsicum, broccoli, kiwifruit, Brussel sprouts, paw paw, cauliflower, orange, mandarin, berries, cabbage, cantaloupe, frozen or mixed vegetables, broad beans, spinach, sweet potato, potato, pineapple, cherries, raw tomato, zucchini, peas, green beans.	Include a fruit or veg high in Vit C: caps, broccoli, kiwi, Brussel sprouts, cauli, orange, mandarin, berries, cabbage, frozen mixed veg, broad beans, spinach, sweet pot, potato, pineapple, cherries, raw tomato, zucchini, peas, green beans

**Fruit**		A variety of vegetables and fruit throughout the menu is important				<u>Variety:</u> (1) At least 2-3 types per day;<br>(2) 5 different types per week	<u>Variety:</u> (1) At least 2-3 types per day;<br>(2) 5 different types per week
			Choose tinned fruit in natural juice, not syrup			Dried fruit not offered more than once per week<br>Fruit bread is not counted towards a serve of fruit	
**Vegetables**		A variety of vegetables and fruit throughout the menu is important	Serve a wide variety of vegetables. Choose canned vegetables and legumes with no added salt			<u>Variety:</u> (1) At least 2-3 types per day;<br>(2) 5 different types per week	<u>Variety:</u> (1) At least 2-3 types per day;<br>(2) 5 different types per week
**Grain (cereal) foods**	High fibre varieties eg multigrain, wholemeal and high fibre white are included daily	High fibre varieties e.g. multigrain, wholemeal, high fibre white are included daily	Choose wholemeal/multigrain varieties where possible		High fibre varieties are included at least 4 times per <u>fortnight</u>	High fibre varieties (e.g. WG & WM) included 3 times per <u>week</u>, preferably every day	High fibre varieties (e.g. WG & WM) included 3 times per <u>week</u>, preferably every day
	Other breads include: pita, English muffins, raisin toast, crumpets.	Other breads include: pita, lavash, Turkish, Lebanese, fruit bread, scones etc.	Include a variety eg pita, Lebanese, fruit loaf		Breads include: loaf, pita, Lebanese and fruit bread		
**Milk, yoghurt, cheese and/or alternatives**		Always use full cream (full fat/regular) cow's milk until the age of 2 years.<br>After 2 years of age, reduced fat (often called "light" or "lite") milk can be used<br><br>Skim milk is not recommended for children until after 5 years of age	Cow or soy milk should not be given before 12 months of age.<br>Reduced fat dairy can be given to children 2 years of age and over.<br>Skim milk can be given to children over 5 years of age<br>Soy milk must be fortified with calcium.<br>Rice and oat milk are not recommended and should only be served to children under medical advice		Full cream milk should be given to children less than 2 years of age<br>Reduced fat and low fat milks (1.0-2.5% fat) are recommended for children over age of 2 years<br>Skim milk (less than 0.5%) should not be used for children under 5 years of age	Full fat varieties should be used for children less than 2 years. Reduced-fat alternatives suitable for children over 2 years	Full fat varieties should be used for children less than 2 years. Reduced-fat alternatives suitable for children over 2 years
	Cream, sour cream and butter are not substitutes for milk, yoghurt and cheese	Cream, sour cream, butte are not substitutes for milk, yoghurt, cheese				Cream, sour cream, butter are not substitutes for milk, yoghurt, and cheese or alternatives	Cream, sour cream, butter are not substitutes for milk, yoghurt, cheese

			The menu should not include sweetened drinks (including flavoured milk			Flavoured milk is not an appropriate drink for young children and should not be offered<br><br>Ensure alternative products (eg soy drinks and soy yoghurt) are calcium fortified with at least 100mg calcium per 100ml	Flavoured milk is not an appropriate drink for young children and should not be included
**Fats and oils**		Ensure the margarines and oils used for cooking are rich in polyunsaturated or monounsaturated fats<br><br>Avoid using fats and oils listed under saturated fat	Preferable to use polyunsaturated or monounsaturated fats		Poly or mono unsaturated oil and/or margarine is used		Poly- or mono-unsaturated oils and spreads used in cooking & baking<br><br>Palm oil, butter, cream … (saturated fats) are not used in cooking
**Salt**							Not added to cooking or available at the table
**Breakfast**			If provided, based on 1 serve breads and cereals and 1 serve dairy eg Weetbix and Milk		If provided, only low sugar breakfast cereals are used - *not further specified*		If provided, based on grains and dairy<br><br>At least one WM or WG choice provided everyday<br>Breakfast cereals are low in added sugars *(less than 15g/100g if dried fruit is not an ingredient; less than 25g/100g if dried fruit is an ingredient)*
**Morning tea (MT) and Afternoon tea (AT)**	Main meals (incl MT and AT) are plotted into menu planning template<br><br>Vegetables and fruit are offered to meet recommended number of serves.<br>Bread/cereal based foods are included if necessary	Midmeals (MT and AT) are planned on the menu as part of the total day's intake<br><br>Milk, cheese, yoghurt or custard is included if necessary to meet the recommended daily serve<br>Bread/cereal based foods are included if necessary	Provide a variety of snack from each food group daily.<br>Do not include discretionary choices.		Snacks for MT, AT are included on the menu each day		Planned and documented on menu<br><br>Based on foods/drinks from 5 food groups

	to meet recommended daily number of serves. Milk, cheese, yoghurt or custard is included if necessary to meet the recommended daily serve of dairy foods.	to meet the recommended daily number of serves Vegetables and fruit are included if necessary to meet the recommended daily number of serves					Baked items are not included everyday
**Late snack**			See 'Cooking for Children and Get up and Grow resource recipes.			…is based on everyday foods and drinks from the five food groups	Based on foods/drinks from 5 food groups
**Drinks**		Soft drinks, sugar sweetened cordials, fruit drinks and sports drinks are not included<br><br>Flavoured milks are not recommended	The menu should not include sweetened drinks (including soft drink, flavoured milk, cordial and sports drinks).<br><br>Flavoured milk not recommended			Soft drinks, fruit drinks, cordial, sports drinks, sports waters, flavoured waters, flavoured mineral waters, iced teas and energy drinks are not offered on the menu<br><br>Flavoured milk and fruit juice are not appropriate drinks for young children and are not offered on the menu	Soft drinks, fruit drinks, sports drinks etc.. are not included<br><br>Flavoured milk not included
**Food variety**		Variety is important.<br><br>Include a variety of textures			Each day the menu offers food of different textures, eg. chewy, soft or minced foods	The menu offers a variety of tastes, colours, textures and flavours<br><br>Main meals are not repeated in a two-week block on the menu<br>Main ingredient in a meal is not repeated on same day each week (e.g. fish every Friday)	Menu includes variety of tastes, colours, textures and flavours<br><br>Main meals are not repeated in a two-week menu cycle<br>Main ingredient in a meal is not repeated on same day each week (e.g. fish every Friday)
**Feeding infants under 12mo**			BM or IF is best for babies under 12 months.<br>Infants under the age of 6 months who are not exclusively breastfed can be offered cooled boiled water in addition to infan formula.		*Policy* lists fluids allowed for birth to age 5	BM, IF, and cooled boiled tap water are the only drinks provided to infants under 12mo	BM, IF, and cooled boiled tap water are the only drinks provided to infants under 12mo

		For children 6 to 12 months, cooled boiled water can supplement BM				
				Policy lists recommend ed stages for introducing solids – *NFS*	From around 6mo, age-appropriate solid foods are offered<br><br>Solid foods are an appropriate texture for infants' developmental stages<br>Iron rich nutritious foods are offered every day	From around 6mo, age-appropriate solid foods are offered<br><br>Solid foods are an appropriate texture for infants' developmental stages<br>Iron rich nutritious foods are offered every day
				Policy documents correct storing/ thawing/warming procedures for BM & IF		
					Fruit, vegetables, grain (cereal) foods and yoghurt or cheese are offered to infants every day in the regular menu	
				*Policy states s*upports breast milk provision and breast feeding mothers		
**Foods and drinks for children with allergies**					Children provided with suitable alternatives to foods and drinks that are not tolerated<br><br>Foods and drinks for children with allergies are documented on the menu	Children provided with suitable alternatives to foods and drinks that are not tolerated<br><br>Foods and drinks for children with allergies are documented on the menu
